# Comparing the clinical and singleton neonatal outcomes in male infertility patients with Oligoasthenospermia, OA, or NOA following fresh ICSI-ET using different sources of sperm

**DOI:** 10.3389/fendo.2023.1186257

**Published:** 2023-11-08

**Authors:** Jianmin Xu, Qingling Yang, Wenhui Chen, Yuqing Jiang, Zhaoyang Shen, Huan Wang, Yingpu Sun

**Affiliations:** ^1^ Center for Reproductive Medicine, The First Affiliated Hospital of Zhengzhou University, Zhengzhou, China; ^2^ Henan Key Laboratory of Reproduction and Genetics, The First Affiliated Hospital of Zhengzhou University, Zhengzhou, China; ^3^ Henan Provincial Obstetrical and Gynecological Diseases (Reproductive Medicine) Clinical Research Center, The First Affiliated Hospital of Zhengzhou University, Zhengzhou, China

**Keywords:** male infertility, sperm, azoospermia, oligospermia, asthenospermia, ICSI

## Abstract

**Objective:**

To investigate clinical and singleton newborn outcomes in fresh cycles of embryo transfer after intracytoplasmic sperm injection (ICSI-ET) with diverse sperm sources (ejaculate, epididymis, and testis) in patients with Oligoasthenospermia, obstructive azoospermia (OA) or non-obstructive azoospermia (NOA).

**Methods:**

Patients who received fresh ICSI-ET for the first time at the First Affiliated Hospital of Zhengzhou University Reproductive Medicine Center between June 2011 and June 2021 were selected for this 10-year retrospective cohort analysis. After propensity score matching, only 1630 cycles were included in the investigation of ICS-ET clinical and singleton newborn outcomes in patients with Oligoasthenospermia, OA, and NOA using sperm from diverse sperm sources.

**Result(s):**

After propensity score matching, our data revealed a negligible difference in baseline and cycle parameters among groups. In patients with Oligoasthenospermia and OA, different sperm sources do not appear to influence clinical pregnancy rates and live birth rates, nor do they influence newborn outcomes, such as newborn weight, premature birth rate, and neonatal sex ratio in singleton births, except for OA patients who use epididymal sperm having higher low birth weight (LBW) rates in singleton pregnancies than those who use testicular sperm. In addition, clinical pregnancy rates, live birth rates, singleton gestation birth weights, premature birth rates, and neonatal sex ratios were similar between patients with Oligoasthenospermia, OA, and NOA using testicular sperm.

**Conclusion(s):**

Regardless of the type of male infertility (Oligoasthenospermia, OA, NOA) or sperm sources (ejaculate, epididymis, testis), a successful ICSI-ET procedure can result in similar clinical and neonatal outcomes, such as clinical pregnancy rate, live birth rate, abortion rate, neonatal birth weight and sex ratio of singleton pregnancies.

## 1 Introduction

Infertility is becoming increasingly recognized as a significant global health issue (1, 2), affecting approximately 15% of married couples, with male infertility accounting for approximately 50% of cases (3–6). Male infertility can be classified into three categories based on sperm count and the presence of sperm disorders: oligospermia, asthenospermia, and azoospermia (7, 8). The latter can further be divided into obstructive azoospermia (OA) and non-obstructive azoospermia (NOA) causes (9).

Initially, donor sperm was the sole option for azoospermic couples. However, the advent of Intracytoplasmic Sperm Injection (ICSI)-Embryo Transfer (ET) in 1992, which involves the surgical retrieval of sperm for fertilization, has allowed couples to have biological offspring (10, 11). Ejaculated, epididymal, and testicular sperm can all be used in ICSI-ET procedures (12). The sperm source depends on the nature of male infertility. Typically, ejaculated sperm is favored in the case of Oligoasthenospermia, epididymal sperm, and testicular sperm can be used in OA cases, and only testicular sperm is used in NOA cases.

However, sperm from these sources differ in maturity, DNA damage, and chromosomal abnormalities, and thus may have implications for the outcomes of ICSI-ET. Testicular sperm have a lower DNA fragmentation index (DFI) than epididymal or ejaculated sperm, suggesting that once sperm are expelled from the testes, they are subjected to oxidative stress and DNA damage (13). Nevertheless, testicular sperm tend to have a greater incidence of chromosomal abnormalities, particularly for sex chromosomes (14), suggesting that even though testicular sperm tend to be preferable to ICSI in terms of DNA damage, this potential benefit may be negated by higher rates of aneuploidy in testicular sperm (15). This raises concerns about the safety of offspring after ICSI-ET and the influence of sperm source and male infertility type on ICSI-ET outcomes (12, 16, 17).

Both the type of male infertility and the source of sperm are potential factors that may impact the outcomes of ICSI-ET. However, few studies have examined the effect of sperm source in combination with male infertility type on ICSI outcomes. This research was designed to investigate clinical and neonatal outcomes of fresh ICSI-ET using ejaculated, epididymal, or testicular sperm in individuals with Oligoasthenospermia, OA, or NOA.

## 2 Materials and methods

### 2.1 Patients selection and classification

Patients who received their first fresh ICSI-ET at the Reproductive Medicine Center of the First Affiliated Hospital of Zhengzhou University from June 2011 to June 2021. The data was obtained from the Clinical Reproductive Medicine Management System/Electronic Medical Records Cohort Database (CCRM/EMRCD) of the Center for Reproductive Medicine. The Ethics Committee of the Hospital authorized this study.

12,984 couples underwent ICSI-ET fresh cycles (excluding proposed PGS/PGD, remedial ICSI, or freeze-thaw embryo transfer), with an initial inclusion of 7,566 cycles which were the first ones using GnRH agonist long protocols. To control for confounding factors, we sequentially excluded oocyte or sperm donation cycles (n=6), karyotype abnormalities in either spouse (n=104), female age ≥ 35 years or < 20 years (n=1456)), female BMI ≥ 28 or ≤ 18 (n=649), female endocrine disorders (PCOS, thyroid disease, diabetes mellitus, hyperprolactinemia) (n=394), female uterine factors affecting implantation and pregnancy (endometriosis, uterine adhesions, multiple fibroids, uterine malformations, endometrial polyps, scarred uterus) (n=412), female infertility factors other than tubal (hypovarianism, immunological factors, benign or malignant gynecological tumors) (n=50), recurrent miscarriages or recurrent implantation failures (n=6), Other male infertility factors (ejaculation disorders, erectile dysfunction, retrograde ejaculation) or cycles with no precise diagnosis (n=622). During the analysis of singleton pregnancy cycles, the exclusion criteria were applied to eliminate cases of multiple pregnancies from the study cohort (n=344).

Based on the type of male infertility and sperm sources, a total of five groups were established: Oligoasthenospermia-Ejaculated sperm group (sperm retrieved from the semen of an Oligoasthenospermic patient), Oligoasthenospermia-Testicular sperm group (sperm recovered from the testis of an Oligoasthenospermic patient), OA-Epididymal sperm group (sperm recovered from the epididymis of an OA patient), OA-Testicular sperm group (sperm recovered from the testis of an OA patient), NOA-Testicular sperm group (sperm recovered from the testis of a patient with NOA).

**Figure 1:**
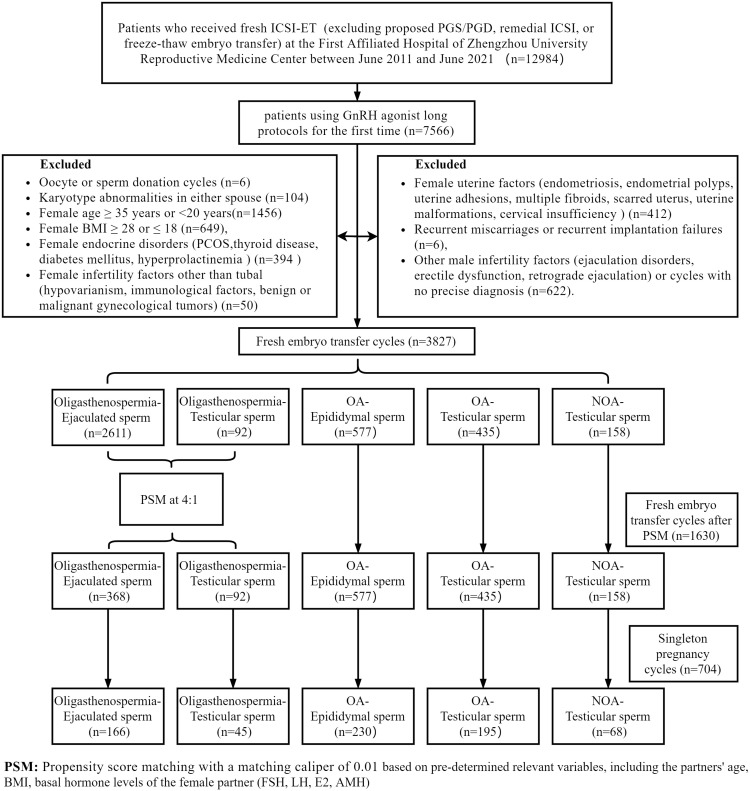
Flow chart of the study design.

To balance the number of cases between groups and to control for confounding factors, propensity score matches were performed without replacement, with a matching caliper of 0.01. Specifically, the Oligoasthenospermia-Ejaculated sperm group was matched 4:1 with the Oligoasthenospermia-Testicular sperm group. Matching was based on pre-determined relevant variables, including the partners’ age, BMI, and basal hormone levels of the female partner (FSH, LH, E2, AMH). At last, the analysis incorporated the remaining 1630 instances. The specific filtering and matching details are shown in **Figure 1**.

### 2.2 Diagnostic criteria

Oligoasthenospermia refers to non-azoospermia with abnormal semen parameters, including Oligozoospermia and Asthenospermia. OA is defined by blockage or partial absence of the reproductive ducts with normal spermatogenic activity due to vasectomy, congenital vas deferens deficiency, or testicular trauma. NOA is characterized by defective spermatogenesis with symptoms ranging from various degrees of maturation stagnation to Sertoli cell-only syndrome.

The diagnostic criteria for Azoospermia are based on the guidelines outlined in the Fifth Edition of the WHO manual for human semen analysis. After ruling out ejaculatory disorders and retrograde ejaculation, a diagnosis of Azoospermia is made when semen is subjected to centrifugation and no sperm is detected through microscopy on more than two occasions. Azoospermia can be further classified into OA and NOA for a more specific diagnosis.

The diagnosis of OA is determined by the following criteria:

1. Normal testicular volume and FSH level.
2. Physical examination reveals a complete epididymis and either an enlarged or absent vas deferens. Reproductive system ultrasound shows dilatation of the epididymal duct or absence of the vas deferens. Seminal plasma biochemistry demonstrates a significant reduction in neutral α-glucosidase.
  Testicular biopsy pathology exhibits a normal basic testicular sperm production function.

NOA is diagnosed when the criteria for OA are not met. Common clinical features of non-obstructive azoospermia include:

1. Testicular volume may be either reduced or normal.
2. FSH levels may be decreased, normal, or elevated.
3. No obstruction of the vas deferens is observed during male physical examination, ultrasound imaging, and seminal plasma biochemistry.
4. Testicular biopsy reveals only supportive cell syndrome or arrested spermatogenesis. In some cases, mature sperm may still be observed.
5. Y chromosome microdeletion is a possible contributing factor.

### 2.3 ICSI

According to the patient’s ovarian function and BMI, gonadotrophin (Gn) was initially administered at doses ranging from 112.5 to 300 IU. Intramuscular hCG (Livzon, China) was administered when transvaginal ultrasound assessment of follicular development revealed that the biggest follicles were > 20 mm in diameter, and more than two-thirds of all follicles had a diameter greater than 16 mm. The retrieval of oocytes was conducted 36-37 h following the hCG injection, and MII oocytes were selected for ICSI 4-6 hours later.

### 2.4 Sperm retrieval

On the day of ovum retrieval, various methods were utilized for obtaining sperm specimens from patients. For patients in the Oligoasthenospermia-Ejaculated sperm group, a 3-7day period of sexual abstinence was observed, followed by semen specimen collection through masturbation and density gradient centrifugation. The obtained sediment was then repeatedly rinsed with sperm washing culture and a 0.5-1.0 mL volume of sperm suspension was retained for ICSI purposes. For patients in the Oligoasthenospermia-Testicular sperm group, the testicular sperm aspiration (TESA) was performed under local anesthesia with 2% lidocaine. The patient was positioned supine and a 5 mL syringe containing 2 mL of culture fluid was utilized to extract testicular tissue under negative pressure. The obtained tissue was mixed and processed through direct centrifugation, and spermatozoa were viewed under an inverted microscope. For patients in the OA-Epididymal sperm group, under local anesthesia with 2% lidocaine, the patient is positioned supine, the Percutaneous epididymal sperm aspiration (PESA) was performed with a 5ml syringe containing 2ml of culture fluid, and epididymal fluid is extracted under negative pressure. For patients in the OA-Testicular sperm group, if no motile sperm were detected following PESA, TESA was conducted as agreed. For patients in the NOA-Testicular sperm group, due to the implementation of testicular sperm extraction (TESE) and microdissection testicular sperm extraction (MD-TESE) technique in our center since November 2018, prior to that, TESA was used to retrieve testicular sperm in NOA patients. Among the 158 NOA patients in this study, 112 underwent TESA, 42 underwent MD-TESE, and 4 underwent TESE. The specific procedure for MD-TESE involved making an incision in the middle of the scrotum under anesthesia, exposing the testis, further dissecting the tunica albuginea, and then, under a microscope (10-20x magnification), observing and selecting the full and opaque seminiferous tubules. Subsequently, the selected tubules were immediately minced by laboratory personnel, and their contents were examined under a microscope (400x magnification) to determine the presence of well-shaped sperm. If no sperm were found, other areas of the testis were examined. If no sperm were found in one testicle, the other testicle was incised, and the same procedure was repeated. Finally, the tunica albuginea and skin incision were sutured. The TESE was performed without microscope and the TSEA surgical procedure was performed as previously described. Approximately 4-6 hours after ovum retrieval, a high-powered microscope was utilized to observe the sperm suspension and select motile sperm with normal morphology for ICSI.

### 2.5 Outcome indicators and definitions

The main outcome measures assessed were the clinical pregnancy rate and live birth rate. Clinical pregnancy was diagnosed through conclusive clinical indications or ultrasound confirmation of at least one gestational sac, including both intrauterine and ectopic pregnancy with medical evidence (18), and live birth rate was a prevalence of births at full 28 weeks following embryo transfer. The secondary observations were biochemical pregnancy and miscarriage rates. In addition, we further analyzed neonatal indicators of singleton pregnancies, including singleton live birth rate (prevalence of births at full 28 weeks following singleton pregnancy), singleton miscarriage rate, neonatal birth weight, gestational age at delivery, neonatal sex, premature birth (a delivery between 28 and 37 weeks), low birth weight (LBW, neonatal birth weight < 2500 g), small for gestational age [SGA, birth weight below the 10th percentile for gestational age; the weight criterion see the latest China Neonatal Weight Survey data (19)], macrosomia (a newborn with a weight over 4,000 g), large for gestational age [LGA, birth weight over the 90th percentile for gestational age, according to the latest China Neonatal Weight Survey data (19)].

### 2.6 Statistical analysis

Statistical analysis and processing were carried out using SPSS 27.0 software. The SPSS missing value Analysis (MVA) procedure with imputation by expectation-maximization (EM) was used for missing data, and propensity score matching was used for case matching. The normality and chi-square tests for continuous variables were One-sample Kolmogorov-Smirnov and Levene tests, respectively. Median (*P*25, *P*75) was used to express continuous variables that were non-normally distributed. Mann-Whitney U test for comparisons between the two groups and Kruskal-Wallis H tests were employed for multiple groups with *post-hoc* Bonferroni corrections following comparisons between two groups. The number of instances and percentages were used to represent categorical variables. The mean of the chi-square value was calculated to analyze differences among groups, and where appropriate, Fisher’s exact tests were performed. Significant differences were defined as *P <* 0.05.

## 3 Result

### 3.1 Baseline and cycle characteristics analysis

**Table 1** presents an analysis of the baseline and cycle characteristics of each group. In patients with Oligoasthenospermia, there was a significant difference in the blastocyst formation rate between the ejaculated sperm group and the testicular sperm group (44.04% vs. 36.76%, *P* = 0.036), with a higher rate observed in the ejaculated sperm group. Among patients with OA, the testicular sperm group had a significantly higher 2PN cleavage rate compared to the epididymal sperm group (98.29% vs. 97.63%, *P* = 0.032). Furthermore, significant differences were observed in several indicators among different groups of male infertility patients using testicular sperm. Firstly, The testicular volume and normal fertilization rate were found to be significantly lower in the NOA group compared to both the Oligoasthenospermia and OA groups, while the blastocyst formation rate in the Oligoasthenospermia group was significantly lower than in the OA and NOA groups. Furthermore, the male basal FSH and LH levels were found to be highest in the NOA group, followed by the Oligoasthenospermia group, whereas the OA group exhibited the lowest levels (*P* < 0.0167). However, there were no statistically significant differences identified in other baseline and cycle characteristics among each group, such as male age and BMI, female age, female basal hormone levels (FSH, E2, LH, P, PRL, T, AMH), female TSH, antral follicle count at trigger timing (AFC), NO. of oocytes retrieved, normal fertilization rate, 2PN cleavage rate, high-quality embryo rate, blastocyst formation rate, NO. of transplanted embryos, and transplanted embryo stage. The results of comparison before and after matching between the Oligoasthenospermia-Ejaculated sperm group and the Oligoasthenospermia-Testicular sperm group were consistent. The data before matching can be found in **Supplementary Table 1**.

**Table 1:** Baseline and cycle characteristics among groups.

	Oligoasthenospermia			OA			NOA	
	Ejaculated sperm (1)	Testicular sperm (2)	*P* 1vs2	Epididymal sperm (3)	Testicular sperm (4)	*P* 3vs4	Testicular sperm (5)	*P* 2vs4vs5
NO. of cycles	368	92		577	435		158	
Male								
Age (year)	29.00 (27.00,32.00)	28.00 (26.00,31.75)	0.211	28.00 (25.00,31.00)	28.00 (26.00,31.00)	0.442	28.00 (26.00,30.25)	0.468
BMI (kg/m²)	24.22 (21.60,27.11)	23.55 (21.21,26.21)	0.315	24.42 (21.96,26.69)	24.54 (22.04,27.04)	0.594	24.49 (22.02,27.68)	0.101
Testicular volume (mL)	12.00 (8.00,14.00)	12.00 (10.00,15.00)	0.168	12.00 (12.00,15.00)	12.00 (10.00,15.00)	0.758	10.00 (8.00,12.00)	***<0.001#***
Basal FSH (IU/L)	6.78 (5.48,10.10)	7.20 (4.73,13.93)	0.126	4.04 (2.93,5.76)	3.99 (2.90,5.51)	0.928	9.55 (4.68,14.87)	<0.001*#
Basal LH (IU/L)	5.37 (3.21,6.83)	5.26 (2.86,6.65)	0.740	4.08 (2.84,5.56)	3.98 (2.83,5.50)	0.600	6.31 (4.45,8.73)	<0.001*#
Basal T (ng/mL)	3.76 (2.98,5.57)	3.64 (3.10,5.08)	0.624	4.07 (2.88,5.43)	4.04 (3.05,5.43)	0.533	4.03 (2.82,5.73)	0.564
Female								
Age (year)	28.00 (26.00,31.00)	28.00 (26.00,30.00)	0.222	27.00 (24.00,29.00)	27.00 (25.00,30.00)	0.060	27.50 (25.00,30.00)	0.306
BMI (kg/m²)	22.02 (20.44,23.88)	22.21 (20.30,23.44)	0.595	21.50 (20.25,23.61)	21.90 (20.31,24.00)	0.443	21.79 (20.20,24.13)	0.932
Basal FSH (IU/L)	6.59 (5.75,7.68)	6.70 (5.72,7.85)	0.672	6.68 (5.74,7.88)	6.54 (5.73,7.64)	0.576	6.53 (5.83,7.58)	0.809
Basal E2 (pg/mL)	36.57 (27.93,48.99)	35.60 (29.51,49.65)	0.538	37.50 (27.41,49.94)	36.84 (27.37,49.21)	0.773	36.86 (29.49,52.48)	0.503
Basal LH (IU/L)	4.90 (3.85,6.30)	4.99 (3.89.6.14)	0.634	4.96 (3.78,6.32)	4.95 (3.83,6.33)	0.995	5.18 (3.58,6.47)	0.974
Basal P (ng/mL)	0.49 (0.31,0.70)	0.45 (0.29,0.72)	0.741	0.50 (0.35,0.75)	0.46 (0.29,0.69)	0.071	0.42 (0.25,0.65)	0.085
Basal PRL (ng/mL)	18.31 (13.82,24.42)	17.29 (12.80,23.70)	0.335	17.86 (12.87,24.48)	17.56 (13.02,23.42)	0.638	17.50 (13.79,21.94)	0.977
Basal T (ng/mL)	0.26 (0.18,0.35)	0.26 (0.17,0.36)	0.705	0.25 (0.18,0.34)	0.26 (0.18,0.36)	0.351	0.29 (0.20,0.39)	0.424
Basal AMH (ng/mL)	3.40 (2.36,4.49)	2.43 (1.46,4.08)	0.288	3.45 (2.38,4.70)	3.30 (2.24,4.55)	0.797	3.06 (2.12,4.05)	0.053
TSH	2.17 (1.57,3.01)	2.26 (1.34,3.21)	0.885	2.09 (1.54,3.10)	2.14 (1.56,2.96)	0.954	2.20 (1.65,3.15)	0.727
No. of oocytes retrieved	13.00 (10.00,16.00)	13.00 (9.25,17.00)	0.472	13.00 (10.00,17.00)	13.00 (10.00,17.00)	0.793	14.00 (10.00,17.00)	0.801
Normal fertilization rate (%)	64.01 (2919/4560)	64.88 (774/1193)	0.579	66.97 (4938/7374)	65.84 (3752/5699)	0.175	62.18 (1320/2123)	***0.011#***
2 PN cleavage rate (%)	98.01 (2861/2919)	97.67 (756/774)	0.555	97.63 (4821/4938)	98.29 (3688/3752)	***0.032***	98.33 (1298/1320)	0.464
High-quality embryo rate (%)	65.85 (1884/2861)	63.23 (478/756)	0.178	64.22 (3096/4821)	63.91 (2357/3688)	0.786	62.56 (812/1298)	0.674
Blastocyst formation rate (%)	44.04 (447/1015)	36.76 (93/253)	***0.036***	48.06 (717/1492)	46.94 (812/1730)	0.526	45.02 (285/633)	0.010*
No. of ET (%)			0.677			0.594		0.670
1	19.29 (71/368)	17.39 (16/92)		22.01 (127/577)	23.22 (101/435)		23.43 (37/158)	
2	80.71 (297/368)	82.61 (76/92)		76.95 (444/577)	76.32 (332/435)		76.58 (121/158)	
3	0.00 (0/368)	0.00 (0/92)		1.04 (6/577)	0.46 (2/435)		0.00 (0/158)	
ET stage (%)			0.746			0.368		0.462
Cleavage stage	84.51 (311/368)	85.87 (79/92)		82.67 (477/577)	80.46 (350/435)		82.28 (130/158)	
Blastocyst stage	15.49 (57/368)	14.13 (13/92)		17.33 (100/577)	19.54 (85/435)		17.72 (28/158)	

Data are provided as medians (P25, P75) for continuous variables and percentages (n/N) for categorical variables.

OA, Obstructive azoospermia; NOA, Non-obstructive azoospermia; BMI, body mass index; FSH, follicle stimulation hormone; E2, estradiol; LH, luteinizing hormone; P, progesterone; PRL, prolactin; AMH, anti-mullerian hormone; AFC, antral follicle count on the hCG trigger day; ET, embryo transferred.

P1vs2, Comparison between the ejaculated sperm group and the testicular sperm group in patients with Oligoasthenospermia; P3vs4, Comparison between the epididymal sperm and testicular sperm groups in patients with OA; P2vs4vs5, Comparison between three groups of Oligoasthenospermia, OA, and NOA using testicular sperm.

P values with significant differences are marked in bold italics. P<0.05. ^#^NOA-Testicular sperm group significant different from the Oligoasthenospermia-Testicular sperm group and the OA-Testicular sperm group. ^*^Significant difference between the Oligoasthenospermia-Testicular sperm group and the OA-Testicular sperm group. The corrected P-value is <0.0167.

### 3.2 ICSI outcomes and singleton neonatal outcomes in Oligoasthenospermic patients with different sperm sources

The study included two Oligoasthenospermia groups, the Oligoasthenospermia-Ejaculated sperm group with 166 singleton gestational cycles in 368 fresh ET cycles and the Oligoasthenospermia-Testicular sperm group with 45 singleton gestational cycles in 92 fresh ET cycles. The fresh transplantation cycle outcomes did not differ between the two groups, including the rates of biochemical pregnancy, clinical pregnancy, live birth, and abortion. Furthermore, the neonatal outcomes of a single pregnancy cycle, including singleton live birth rate, singleton abortion rate, neonatal birth weight, gestational age at delivery, neonatal sex ratio, and preterm birth rate, also did not show any significant differences between the two groups. Furthermore, we conducted detailed analyses of neonatal health based on both gestational week and birth weight and found no significant difference in LBW, SGA, macrosomia, and LGA. Details of specific data are provided in **Table 2**. Additionally, the results of the unmatched analysis did not reveal any differences, as shown in **Supplementary Table 2**.

**Table 2:** ICSI outcomes and singleton neonatal outcomes in Oligoasthenospermic patients with different sperm sources.

	Oligoasthenospermia		
	Ejaculated sperm	Testicular sperm	*P*
First fresh ET cycles:			
NO. of cycles	368	92	
Biochemical pregnancy rate (%)	73.64 (271/368)	75.00 (69/92)	0.791
Clinical pregnancy rate (%)	69.57 (256/368)	67.39 (62/92)	0.686
Live birth rate (%)	61.14 (225/368)	60.87 (56/92)	0.962
Miscarriage rate (%)	7.34 (27/368)	5.43 (5/92)	0.521
Singleton pregnancy cycles:			
No. of cycles	166	45	
Singleton live birth rate (%)	93.37 (155/166)	93.33 (42/45)	1.000
Singleton abortion rate (%)	5.42 (9/166)	6.67 (3/45)	1.000
Neonatal birth weight (g)	3400.00 (3100.00, 3600.00)	3450.00 (3000.00-3700.00)	0.803
Gestational weeks at delivery (week)	39.00 (38.00, 40.00)	39.00 (38.00-40.00)	0.452
Neonatal sex (%)			0.159
Male	50.32 (78/155)	38.10 (16/42)	
Female	49.68 (77/155)	61.91 (26/42)	
Preterm birth (%)	5.81 (9/155)	2.38 (1/42)	0.616
Low birth weight (%)	2.58 (4/155)	2.38 (1/42)	1.000
Small for gestational age (%)	6.45 (10/155)	9.52 (4/42)	0.727
Macrosomia (%)	7.74 (12/155)	11.91 (5/42)	0.367
Large for gestational age (%)	19.35 (30/155)	23.81 (10/42)	0.524

Data are provided as percentages (n/N) for categorical variables.

### 3.3 ICSI and singleton neonatal outcomes in OA patients with different sperm sources

In the OA-Epididymal sperm group, 230 singleton gestational cycles occurred in 577 fresh ET cycles, whereas in the OA-Testicular sperm group, 195 singleton gestational cycles occurred in 435 fresh ET cycles. A comparison of fresh ICSI-ET results showed no significant intergroup differences, including the rates of biochemical pregnancy, clinical pregnancy, live birth, and abortion. When it comes to the neonatal outcome of a single pregnancy cycle, the only noteworthy difference between the OA-Testicular sperm and OA-Epididymal sperm groups is the LBW (1.10% vs. 5.16%, *P =* 0.024). However, further detailed analysis of gestational weeks and neonatal birth weight revealed no difference in gestational weeks, premature birth rate, and SGA. In addition, both groups showed no noticeable differences in singleton live birth rate, abortion rate, neonatal birth weight, neonatal sex ratio, macrosomia, or LGA. Detailed data are shown in **Table 3**.

**Table 3:** ICSI and singleton neonatal outcomes in OA patients with different sperm sources.

		OA	
	Epididymal sperm	Testicular sperm	*P*
First fresh ET cycles:			
NO. of cycles	577	435	
Biochemical pregnancy rate (%)	69.84 (403/577)	75.40 (328/435)	0.051
Clinical pregnancy rate (%)	66.72 (385/577)	71.36 (310/435)	0.123
Live birth rate (%)	59.10 (341/577)	63.33 (275/435)	0.184
Miscarriage rate (%)	6.41 (37/577)	6.90 (30/435)	0.759
Singleton pregnancy cycles:			
No. of cycles	230	195	
Singleton live birth rate (%)	92.61 (213/230)	93.33 (182/195)	0.771
Singleton abortion rate (%)	5.65 (13/230)	6.67 (13/195)	0.771
Neonatal birth weight (g)	3350.00 (3100.00-3650.00)	3400.00 (3150.00-3687.50)	0.931
Gestational weeks at delivery (week)	39.00 (38.00-40.00)	39.00 (38.00-40.00)	0.511
Neonatal sex (%)			0.912
Male	51.64 (110/213)	52.20 (95/182)	
Female	48.36 (103/213)	47.80 (87/182)	
Preterm birth (%)	5.16 (11/213)	4.95 (9/182)	0.921
Low birth weight (%)	5.16 (11/213)	1.10 (2/182)	***0.024***
Small for gestational age (%)	7.98 (17/213)	6.04 (11/182)	0.455
Macrosomia (%)	7.98 (17/213)	9.34 (17/182)	0.631
Large for gestational age (%)	18.78 (40/213)	22.53 (41/182)	0.358

Data are provided as medians (P25, P75) for continuous variables and percentages (n/N) for categorical variables.

P values with significant differences are marked in bold italics, p<0.05.

### 3.4 ICSI and singleton neonatal outcomes based on the type of male infertility using testicular sperm

A cohort of 685 patients using testis sperm for ICSI, which was further divided into three groups: Oligoasthenospermia (n=92), OA (n=435), and NOA (n=158). The analysis of fresh ICSI-ET cycles showed no significant differences between the three groups in terms of biochemical pregnancy rate, clinical pregnancy rate, live birth rate, or abortion rate. The neonatal outcomes of a single pregnancy cycle were analyzed and showed that there were 45 singleton gestational cycles in the Oligoasthenospermia group, 195 singleton gestational cycles in the OA-Testicular sperm group, and 68 singleton gestational cycles in the NOA-Testicular sperm group. The analysis of these groups revealed no noticeable differences in singleton live birth rate, singleton abortion rate, neonatal birth weight, gestational age at delivery, neonatal sex ratio, premature birth rate, LBW, SGA, macrosomia, or LGA. Detailed data can be found in **Table 4**.

**Table 4:** ICSI and singleton neonatal outcomes based on the type of male infertility using testicular sperm.

	Testicular sperm			
	Oligoasthenospermia	OA	NOA	*P*
First fresh ET cycles:				
NO. of ycles	92	435	158	
Biochemical pregnancy rate (%)	75.00 (69/92)	75.40 (328/435)	72.78 (115/158)	0.809
Clinical pregnancy rate (%)	67.39 (62/92)	71.36 (310/435)	66.46 (105/158)	0.468
Live birth rate (%)	60.87 (56/92)	63.33 (275/435)	59.49 (94/158)	0.689
Miscarriage rate (%)	5.43 (5/92)	6.90 (30/435)	5.06 (8/158)	0.674
Singleton pregnancy cycles:				
No. of cycles	45	195	68	
Singleton live birth rate (%)	93.33 (42/45)	93.33 (182/195)	91.18 (62/68)	0.811
Singleton abortion rate (%)	6.67 (3/45)	6.67 (13/195)	7.35 (5/68)	0,947
Neonatal birth weight (g)	3450.00 (3000.00-3700.00)	3400.00 (3150.00-3687.50)	3350.00 (3050.00-3650.00)	0.123
Gestational weeks at delivery (week)	39.00 (38.00-40.00)	39.00 (38.00-40.00)	39.00 (38.00-40.00)	0.395
Neonatal sex (%)				0.227
Male	38.10 (16/42)	52.20 (95/182)	51.61 (32/62)	
Female	61.91 (26/42)	47.80 (87/182)	48.39 (30/62)	
Preterm birth (%)	2.38 (1/42)	4.95 (9/182)	6.45 (4/62)	0.725
Low birth weight (%)	2.38 (1/42)	1.10 (2/182)	4.84 (3/62)	0.109
Small for gestational age (%)	9.52 (4/42)	6.04 (11/182)	3.23 (5/62)	0.588
Macrosomia (%)	7.14 (3/42)	9.34 (17/182)	9.68 (6/62)	0.890
Large for gestational age (%)	23.81 (10/42)	22.53 (41/182)	26.83 (17/62)	0.737

Data are provided as medians (P25, P75) for continuous variables and percentages (n/N) for categorical variables.

## 4 Discussion

In this study, different male infertility populations and different sources of sperm were distinguished to evaluate their effects on ICSI-ET clinical outcomes and neonatal outcomes. Our data reveal that although normal fertilization and blastocyst formation rates differed to varying degrees between groups before the transfer, different male infertility populations and different sources of sperm did not appear to affect ICSI clinical and singleton neonatal outcomes after successful transfer.

Regarding the research on ART and neonatal outcomes using ejaculated sperm versus testicular sperm. Fedder J et al. (20) reported a significantly lower sex ratio in the offspring of ICSI using epididymal or testicular sperm compared to conventional IVF. Our study also revealed a decreased sex ratio in cycles utilizing testicular sperm compared to cycles involving ejaculated sperm, particularly in patients diagnosed with Oligoasthenospermia, despite the lack of a statistical difference (38.10% vs. 50.12%, *P* > 0.05). In addition, another cohort study (21), with consistent findings to our research, indicated that children born as singletons or twins following ICSI using epididymal or testicular sperm exhibited comparable perinatal or neonatal outcomes to those born after IVF and ICSI using ejaculated sperm. However, there are studies that present contradictory results to our research findings. For instance, Lauren M et al. (22) reported ICSI outcomes in non-azoospermic patients using ejaculated sperm (n=32) and TESE-derived sperm and found that sperm retrieved using TESE had an apparent decrease in the live birth rate (31.3% vs. 50.0%, *P >* 0.05), although the difference did not reach statistical significance. Contrary to their findings, the results of our study indicated that the live birth rates of ejaculated sperm and Testicular sperm after ICSI-ET were similar. Moreover, a study conducted by Rene H et al. (23) examined 27 couples who initially attempted ICSI with ejaculated sperm but did not achieve a successful pregnancy. Subsequently, they switched to using testicular sperm, and the study revealed that this shift significantly improved blastocyst quality without increasing the risk of aneuploidy. However, our results indicated that among patients with Oligoasthenospermia, the cycles utilizing testicular sperm demonstrated a significantly lower blastocyst formation rate compared to the cycles using ejaculated sperm.

Regarding the study on the results of ICSI using epididymal or testicular sperm in patients with OA. Buffat C et al. (24) and Semiao-Francisco L et al. (25) reported comparable clinical pregnancy and live birth rates in OA patients using PESA-derived sperm versus TESA-derived sperm despite differences in fertilization and miscarriage rates. Jin L et al. (26) reported that epididymal sperm obtained comparable neonatal outcomes to testicular sperm. A meta-analysis involving eight studies observed no notable disparities in rates of miscarriage and clinical pregnancy after ICSI whether TESA- or PESA-derived sperm were used (27). Our study corroborates the conclusions of these previous studies as we also found no significant difference in rates of clinical pregnancy, live birth, or miscarriage between the groups using epididymal sperm and testicular sperm in patients with OA. However, our study also found that the 2PN cleavage rate was significantly higher in the OA-testicular sperm group compared to the OA-epididymal sperm group, while the LBW rate was significantly lower in the OA-testicular sperm group compared to the OA-epididymal sperm group.

Regarding the results of ICSI using testicular sperm in various cases of male infertility. Our study found that patients with Oligoasthenospermia, OA, or NOA using testicular sperm achieved similar clinical and neonatal outcomes. Several similar studies have reached consistent conclusions with ours. Igael Madgar et al. (28) compared 1103 cycles in OA patients to 275 cycles in NOA patients without distinguishing the sperm source and reported comparable clinical pregnancy rates between the two groups, indicating no significant difference. Similarly, another research evaluated the clinical results of different populations using TESA-derived sperm. Despite their limited comparison of OA and NOA, they discovered that the rates of normal fertilization and implantation, as well as clinical pregnancy, were comparable (25). Furthermore, Sadek S et al. (29) found that the ART and perinatal outcomes of NOA and OA couples using testicular sperm were comparable to those of couples with Male Factor infertility, with success primarily depending on the age of the patient and their partner.

Our study results show that despite differences in rates of fertilization and embryo development when using sperm from different sources in males with infertility, similar outcomes were observed regarding clinical pregnancy rates, live birth rates, and neonatal outcomes. This may be attributed to the inherent mechanisms of ICSI, which overrides several critical steps in the traditional sperm-egg unions process, such as sperm capacitation, acrosome reaction, and recognition of the zona pellucida. By directly injecting sperm into the oocyte cytoplasm, ICSI greatly reduces the requirement for high sperm count and motility. Additionally, since only one sperm is needed during ICSI, the chances of fertilization for males with infertility remain relatively consistent regardless of whether the sperm is obtained from ejaculated semen, epididymis, or testes. Furthermore, the selection and transfer of high-quality embryos during the embryo culture stage may also play a role in mitigating the impact of sperm quality differences.That is, even though sperm obtained from different sperm retrieval methods and different male infertility patients have different levels of malformation, DNA fragmentation, acrosome integrity, and maturity, the ICSI-ET procedure may have effectively mitigated these differences through the selection process.

To our knowledge, this was the first time that patients with Oligoasthenospermia using testicular sperm were simultaneously being compared with OA and NOA patients. Additionally, strict inclusion-exclusion criteria and propensity score matching were conducted beforehand in our investigation to balance confounding factors and promote comparability. Furthermore, the sample size in our research was greater than most previous studies on this topic. However, it should be noted that the study has some limitations. Firstly, being a retrospective study, it is susceptible to selection bias. Moreover, factors such as medication usage and environmental variables related to male infertility were not considered in the study, which may have affected the results. Lastly, considering the ten-year duration of this study and the continuous advancements in clinical diagnostics and treatments, prior to November 2018, NOA patients were exclusively subjected to TESA for sperm retrieval. Subsequently, some patients began to utilize MD-TESE and TESE techniques, potentially contributing to result heterogeneity. Despite these limitations, our study has unique contributions to the field. By strictly differentiating the male infertility patient population, we were able to compare ICSI-ET clinical and neonatal outcomes three times systematically, providing insights into the differences between different sperm sources and male infertility patients.

In summary, the preliminary conclusion of our study indicates that regardless of the type of male infertility (Oligoasthenospermia, OA, NOA) or sperm source (ejaculate, epididymis, testis), a successful ICSI-ET procedure can result in similar clinical and neonatal outcomes, such as the rates of clinical pregnancy, live birth, and miscarriage, neonatal birth weight and sex ratio of singleton pregnancies. We anticipate that our findings will provide male infertility patients and physicians some peace of mind when deciding on the best sperm retrieval procedure and that larger multicentre studies incorporating more patients will be conducted to validate further and complement our findings.

## Data availability statement

The raw data supporting the conclusions of this article will be made available by the authors, without undue reservation.

## Ethics statement

The studies involving humans were approved by Ethics Committee of the First Affiliated Hospital of Zhengzhou University. The studies were conducted in accordance with the local legislation and institutional requirements. Written informed consent for participation was not required from the participants or the participants’ legal guardians/next of kin in accordance with the national legislation and institutional requirements.

## Author contributions

JX selected the population to be included and excluded, completed data statistics and analysis and wrote the manuscript. QY participated in the research design, guidance, and manuscript revision. YS participated in the review and guidance of the research. WC, YJ, ZS, and HW participated in data entry and sorting. JX and QY contributed equally to this article. All authors contributed to the article and approved the submitted version.
