# Supplementary material for: Comparing the clinical and singleton neonatal outcomes in male infertility patients with Oligoasthenospermia, OA, or NOA following fresh ICSI-ET using different sources of sperm
Source: Front Endocrinol (Lausanne). 2023 Nov 8;14:1186257. doi: 10.3389/fendo.2023.1186257 (PMC10663326; doi:10.3389/fendo.2023.1186257)
Supplement: Supplementary file 1 [file Table_1.docx]

Supplementary Material

Comparing the Clinical and Singleton Neonatal Outcomes in Male Infertility Patients with Oligoasthenospermia, OA, or NOA Following Fresh ICSI-ET Using Different Sources of Sperm.

**Jianmin Xu^1 2 3 †^, Qingling Yang^1 2 3 †^, Wenhui Chen^1 2 3^, Yuqing Jiang^1 2 3^, Zhaoyang Shen^1 2 3^, Huan Wang^1 2 3^, Yingpu Sun^1 2 3 *^**

*** Correspondence:** Corresponding Author: Yingpu Sun, Email: [syp2008@vip.sina.com](mailto:syp2008@vip.sina.com)

† These authors have contributed equally to this work.

**Supplementary Table 1: Baseline and cycle characteristics of Oligoasthenospermic patients with different sperm sources before PSM.**

|  | Oligoasthenospermia | |  |
| --- | --- | --- | --- |
|  | Ejaculated sperm | Testicular sperm | *P* |
| NO. of cycles | 2611 | 92 |  |
| Male |  |  |  |
| Age（year） | 29.00(27.00,32.00) | 28.00(26.00,31.75) | 0.566 |
| BMI（kg/m²） | 24.49(22.09,26.99) | 23.55(21.21,26.21) | 0.134 |
| Testicular volume(mL) | 12.00(9.00,14.00) | 12.00(10.00,15.00) | 0.254 |
| Basal FSH(IU/L) | 6.92(5.64,10.50) | 7.20(4.73,13.93) | 0.198 |
| Basal LH(IU/L) | 5.41(3.14,6.92) | 5.26(2.86,6.65) | 0.560 |
| Basal T(ng/mL) | 3.66(2.86,5.32) | 3.64(3.10,5.08) | 0.876 |
| Female |  |  |  |
| Age（year） | 28.00(26.00,31.00) | 28.00(26.00,30.00) | 0.480 |
| BMI（kg/m²） | 22.00(20.40,23.90) | 22.21(20.30,23.44) | 0.739 |
| Basal FSH(IU/L) | 6.58(5.62,7.70) | 6.70(5.72,7.85) | 0.749 |
| Basal E2(pg/mL) | 37.21(28.00,49.56) | 35.60(29.51,49.65) | 0.383 |
| Basal LH(IU/L) | 4.83(3.59,6.27) | 4.99（3.89.6.14） | 0.383 |
| Basal P (ng/mL) | 0.49(0.31,0.71) | 0.45(0.29,0.72) | 0.854 |
| Basal PRL (ng/mL) | 17.50(12.95,24.30) | 17.29(12.80,23.70) | 0.965 |
| Basal T(ng/mL) | 0.26(0.18,0.35) | 0.26(0.17,0.36) | 0.794 |
| Basal AMH(ng/mL) | 3.08(2.09,4.38) | 2.43(1.46,4.08) | 0.065 |
| TSH | 2.14(1.52,3.04) | 2.26(1.34,3.21) | 0.452 |
| AFC | 28.00(26.00,31.00) | 28.00(26.00,30.00) | 0.480 |
| No. of oocytes retrieved | 13.00(9.00,17.00) | 13.00(9.25,17.00) | 0.848 |
| Normal fertilization rate (%) | 71.47(19234/26911) | 70.45(739/1049) | 0.471 |
| 2 PN cleavage rate (%) | 98.46(20393/20713) | 97.67(478/774) | 0.087 |
| High-quality embryo rate (%) | 64.60(13173/20393) | 63.23(478/756) | 0.440 |
| Blastocyst formation rate (%) | 47.57(3688/7753) | 36.76(93/253) | ***0.001*** |
| No. of ET(%) |  |  | 0.527 |
| 1 | 20.57(537/2611) | 17.39(16/92) |  |
| 2 | 78.71(2055/2611) | 82.61(76/92) |  |
| 3 | 0.73(19/2611) | 0.00(0/92) |  |
| ET stage(%) |  |  | 0.486 |
| Cleavage stage | 83.11(2170/2611) | 85.87(79/92) |  |
| Blastocyst stage | 16.89(441/2611) | 14.13(13/92) |  |

Data are provided as medians (*P*25, *P*75) for continuous variables and percentages (n/N) for categorical variables.

Note for abbreviations: OA, Obstructive azoospermia; NOA, Non-obstructive azoospermia; TESA, testicular sperm aspiration; PESA, percutaneous epididymal sperm aspiration; BMI, body mass index; FSH, follicle stimulation hormone; E2, estradiol; LH, luteinizing hormone; P, progesterone; PRL, prolactin; AMH, anti-mullerian hormone; AFC, antral follicle count on the hCG trigger day; ET, embryo transferred.

*P* values with significant differences are marked in bold italics, *P<0.05*.

**Supplementary Table 2: Clinal and neonatal outcomes of in Oligoasthenospermic patients with different sperm sources before PSM.**

|  | Oligoasthenospermia | |  |
| --- | --- | --- | --- |
|  | Ejaculated sperm | Testicular sperm | *P* |
| **First fresh ET cycles:** |  |  |  |
| NO. of fresh ET cycles | 2611 | 92 |  |
| Biochemical pregnancy rate (%) | 69.67(1819/2611) | 75.00(69/92) | 0.273 |
| Clinical pregnancy rate (%) | 65.99(1723/2611) | 67.39(62/92) | 0.78 |
| Live birth rate (%) | 58.48(1527/2611) | 60.87(56/92) | 0.648 |
| Miscarriage rate (%) | 6.55(171/2611) | 5.43(5/92) | 0.670 |
| **Singleton pregnancy cycles:** |  |  |  |
| No. of cycles | 1109 | 45 |  |
| Singleton live birth rate (%) | 93.60(1038/1109) | 93.33(42/45) | 1.000 |
| Singleton abortion rate (%) | 5.59(62/1109) | 6.67(3/45) | 1.000 |
| Neonatal birth weight (g) | 3400.00(3100.00-3700.00) | 3450.00(3000.00-3700.00) | 0.500 |
| Gestational weeks at delivery (week) | 39.00(38.00-40.00) | 39.00(38.00-40.00) | 0.988 |
| Neonatal sex (%) |  |  | 0.161 |
| Male | 49.13(510/1038) | 38.10(16/42) |  |
| Female | 50.87(528/1038) | 61.91(26/42) |  |
| Preterm birth(%) | 3.94(40/1015) | 2.38(1/42) | 0.916 |
| Low birth weight(%) | 2.47(25/1012) | 2.38(1/42) | 1.000 |
| Small for gestational age (%) | 5.34(54/1012) | 9.52(4/42) | 0.412 |
| Macrosomia (%) | 12.25(124/1012) | 11.91(5/42) | 0.946 |
| Large for gestational age (%) | 23.62(239/1012) | 23.81(10/42) | 0.977 |

Data are provided as medians (*P*25, *P*75) for continuous variables and percentages (n/N) for categorical variables.
